# Genome-wide Association Study for AKI

**DOI:** 10.34067/KID.0000000000000175

**Published:** 2023-06-05

**Authors:** Pavan K. Bhatraju, Ian B. Stanaway, Melody R. Palmer, Rajasree Menon, Jennifer A. Schaub, Steven Menez, Anand Srivastava, F. Perry Wilson, Krzysztof Kiryluk, Paul M. Palevsky, Abhijit S. Naik, Sana S. Sakr, Gail P. Jarvik, Chirag R. Parikh, Lorraine B. Ware, T. Alp Ikizler, Edward D. Siew, Vernon M. Chinchilli, Steve G. Coca, Amit X. Garg, Alan S. Go, James S. Kaufman, Paul L. Kimmel, Jonathan Himmelfarb, Mark M. Wurfel

**Affiliations:** 1 Division of Pulmonary, Critical Care and Sleep Medicine, Department of Medicine, University of Washington, Seattle, Washington; 2 Kidney Research Institute, Division of Nephrology, Department of Medicine, University of Washington, Seattle, Washington; 3 Departments of Medicine (Medical Genetics) and Genome Sciences, University of Washington School of Medicine, Seattle, Washington; 4 Division of Nephrology, Department of Medicine, Michigan Medicine, Ann Arbor, Michigan; 5 Division of Nephrology, Johns Hopkins University School of Medicine, Baltimore, Maryland; 6 Department of Medicine, Division of Nephrology and Hypertension, Northwestern University School of Medicine, Chicago, Illinois; 7 Program of Applied Translational Research, Yale School of Medicine, New Haven, Connecticut; 8 Division of Nephrology, Department of Medicine, Vagelos College of Physicians & Surgeons, Columbia University, New York City, New York; 9 Kidney Medicine Section, VA Pittsburgh Healthcare System, Pittsburgh, Pennsylvania; 10 Renal-Electrolyte Division, Department of Medicine, University of Pittsburgh School of Medicine, Pittsburgh, Pennsylvania; 11 Division of Nephrology, University of Michigan, Ann Arbor, Michigan; 12 Division of Allergy, Pulmonary and Critical Care, Vanderbilt University Medical Center, Nashville, Tennessee; 13 Division of Nephrology and Hypertension, Vanderbilt University Medical Center, Nashville, Tennessee; 14 Department of Public Health Sciences, Penn State College of Medicine, Hershey, Pennsylvania; 15 Section of Nephrology, Department of Internal Medicine, Mount Sinai School of Medicine, New York, New York; 16 Division of Nephrology, Department of Medicine, Western University, London, Ontario, Canada; 17 Division of Nephrology, Department of Medicine, University of California, San Francisco, California; 18 Division of Research, Kaiser Permanente Northern California, Oakland, California; 19 Department of Epidemiology and Biostatistics, University of California, San Francisco, California; 20 Division of Nephrology, New York University School of Medicine, New York, New York; 21 Division of Nephrology, VA New York Harbor Healthcare System, New York, New York; 22 Division of Renal Diseases and Hypertension, Department of Medicine, George Washington University Medical Center, Washington, DC

**Keywords:** AKI, human genetics, molecular genetics

## Abstract

**Key Points:**

- Two genetic variants in the DISP1-TLR5 gene locus were associated with risk of AKI.
- DISP1 and TLR5 were differentially regulated in kidney biopsy tissue from patients with AKI compared with no AKI.

**Background:**

Although common genetic risks for CKD are well established, genetic factors influencing risk for AKI in hospitalized patients are poorly understood.

**Methods:**

We conducted a genome-wide association study in 1369 participants in the Assessment, Serial Evaluation, and Subsequent Sequelae of AKI Study; a multiethnic population of hospitalized participants with and without AKI matched on demographics, comorbidities, and kidney function before hospitalization. We then completed functional annotation of top-performing variants for AKI using single-cell RNA sequencing data from kidney biopsies in 12 patients with AKI and 18 healthy living donors from the Kidney Precision Medicine Project.

**Results:**

No genome-wide significant associations with AKI risk were found in Assessment, Serial Evaluation, and Subsequent Sequelae of AKI (*P < 5×10*^*−8*^). The top two variants with the strongest association with AKI mapped to the *dispatched resistance-nodulation-division (RND) transporter family member 1 (DISP1)* gene and *toll-like receptor 5 (TLR5)* gene locus, rs17538288 (odds ratio, 1.55; 95% confidence interval, 1.32 to 182; *P = 9.47×10*^*−8*^) and rs7546189 (odds ratio, 1.53; 95% confidence interval, 1.30 to 1.81; *P = 4.60×10*^*−7*^). In comparison with kidney tissue from healthy living donors, kidney biopsies in patients with AKI showed differential *DISP1* expression in proximal tubular epithelial cells (adjusted *P* = 3.9*×*10^−2^) and thick ascending limb of the loop of Henle (adjusted *P* = 8.7*×*10^−3^) and differential *TLR5* gene expression in thick ascending limb of the loop of Henle (adjusted *P* = 4.9*×*10^−30^).

**Conclusions:**

AKI is a heterogeneous clinical syndrome with various underlying risk factors, etiologies, and pathophysiology that may limit the identification of genetic variants. Although no variants reached genome-wide significance, we report two variants in the intergenic region between *DISP1* and *TLR5*, suggesting this region as a novel risk for AKI susceptibility.

## Graphical abstract

**Figure:**
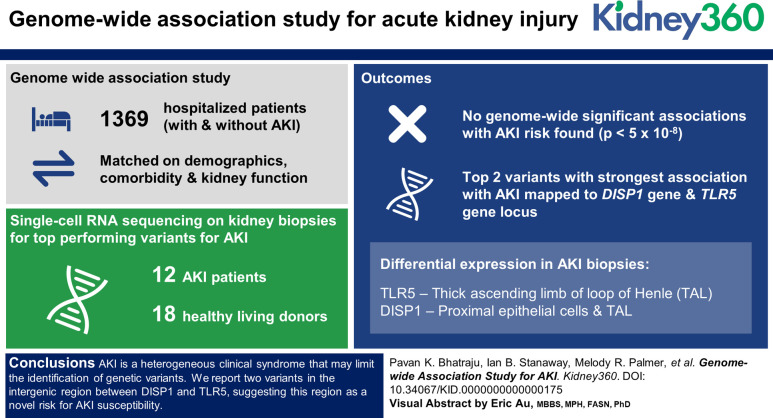

## Introduction

AKI affects approximately 20% of hospitalized patients,^1^ and 40% of patients admitted to the intensive care unit^2^ and contributes significantly to poor long-term outcomes.^3–5^ Despite this public health effect, no effective pharmacotherapy exists for AKI.^6^ An approach identifying genetic risk factors for AKI could enhance efforts to develop novel preventive and therapeutic strategies. A genetic predisposition for AKI has been suggested by previous candidate gene studies and two prior genome-wide association studies (GWAS).^7,8^ However, limitations in power due to sample size and analyses completed in predominantly European ancestry (EA) populations may have restricted identification of genetic variants for the development of AKI.^9,10^ Another limitation of conducting genetic association studies for AKI in hospitalized cohorts is the issue of competing risk.^11^ Genetic cohorts of hospitalized patients, particularly critically ill patients, can experience in-hospital mortality of 20%–60%^8,12^ which could prevent the occurrence of organ failure outcomes, such as the development of AKI, that require some period of observation to ascertain. A final limitation is the difficulties in obtaining an outpatient baseline serum creatinine for accurate diagnosis of AKI.^13,14^ The Kidney Disease Improving Global Outcomes (KDIGO) consensus group defines AKI as an increase in serum creatinine of ≥0.3 mg/dl from the baseline within a 48-hour period or an increase in serum creatinine of ≥50% from the baseline within 7 days.^15^ This definition relies on an adequate baseline or outpatient measure of kidney function before hospitalization, which is often lacking in hospitalized populations.

To account for issues of timing, severity of AKI, and competing risks, we prospectively enrolled a multiethnic population for genotyping in the Assessment, Serial Evaluation, and Subsequent Sequelae of AKI (ASSESS-AKI) Study.^16–18^ All ASSESS-AKI participants had a baseline outpatient serum creatinine before hospitalization, and AKI cases were matched to non-AKI controls on the basis of baseline demographics and comorbidities. In addition, all participants enrolled in ASSESS-AKI had to survive the index hospitalization. Thus, ASSESS-AKI improves the precision in the AKI phenotype and minimizes the competing risk of early death making it a unique cohort to study genetic risk factors for the development of AKI. Given that multiethnic cohorts with genomic data similar to ASSESS-AKI currently do not exist we sought other ways to functionally validate our findings. We identified the precise cell types that express candidate genes associated with AKI in ASSESS-AKI and the degree to which these genes were differentially expressed in AKI using a single-cell Ribonucleic acid sequencing (RNAseq) dataset from AKI biopsy tissue obtained through the Kidney Precision Medicine Project (KPMP).^19^ Some of the results of these studies have been previously reported in abstract form.^20^

## Methods

### Study Populations

We performed a GWAS of AKI in 1369 adults in ASSESS-AKI who consented to genetic analyses and for whom genotyping passed quality measures. The full ASSESS-AKI cohort included 1538 hospitalized adults who did or did not experience an episode of AKI and survived to complete a study visit 90 days after discharge.^16,21^ Adults with AKI were matched to hospitalized adults without AKI on the basis of site, preadmission CKD status, age, prior cardiovascular disease, diabetes mellitus, and preadmission eGFR using the Chronic Kidney Disease Epidemiology Collaboration equation. This cohort is racially/ethnically diverse, with representation from non-Hispanic White, Hispanic/Latino, East Asian, and African American race/ethnicity groups. All study procedures were approved by the local Institutional Review Boards.

### AKI Definition

In ASSESS-AKI, AKI cases were defined using modified KDIGO criteria on the basis of an increase of ≥50% or ≥0.3 mg/dl in serum creatinine above a baseline serum creatinine value obtained at an outpatient, nonemergency department measurement between 7 and 365 days before the index admission.^21^ Urine output was not used to define AKI.

### Genotyping and Imputation

In ASSESS-AKI, genotyping was performed in one batch, using the Illumina Multiethnic Global Array platform. Samples with a call rate ≥97% were included in the analyses. No participants were removed for genotyping quality. Two participants were removed for discordance between genetic sex and self-reported sex. Another participant from a pair of participants identified with cryptic relatedness was removed. Genome-wide variant imputation was conducted with EAGLE v2.3^22^ phasing on the Michigan Imputation Server^23^ using the Haplotype Reference Consortium r1.1 2016.^24^ We included genetic variants or single-nucleotide polymorphisms (variants) with an imputation quality r^2^ >0.3, Hardy–Weinberg Equilibrium *P* > 10^−6^ in the EA participants, and a minor allele frequency >1% for a final variant count of 8,669,569.

Two variants with the strongest associations were then selected for direct genotyping using RT-PCR methods. Direct genotyping of rs17538288 and rs7546189 was performed with TaqMan assays using the QuantStudio 7 Real-time PCR System (Thermo Fisher). Lymphoblastoid cell lines derived from participants in the 1000 Genomes Project were used as controls. We achieved a genotype call rate of 98.7% (1471/1490) for rs17538288 and 100% (1456/1456) for rs7546189, with complete agreement between duplicates, and expected genotypes for the 1000 genomes samples. The results did not deviate significantly from Hardy–Weinberg Equilibrium.

### Statistical Analysis

Patient demographic variables are reported as either mean and standard deviation or median and quartiles. The primary end point for our GWAS was the development of AKI. Odds ratios (ORs) are reported with 95% confidence intervals (CIs). We tested in logistic regression models for AKI using an additive genetic model with the imputed variants while conditioning on the following covariates: participant's age, sex, diabetes, hypertension, study site enrollment, and ten principal components (PCs) of ancestry. In a sensitivity analysis, we tested whether the top variants were associated with severity of kidney injury modeling AKI status as an ordinal variable (0=no AKI, 1=KDIGO Stage 1 AKI, 2=KDIGO Stage 2, and 3=KDIGO Stage 3). Additional *post hoc* regressions were performed with R statistical software to evaluate the extent to which associations between variants and AKI at a particular locus were independent. We also performed compound heterozygosity analysis.^25^ In the *post hoc* tests, the RT-PCR genotype results of the two most associated variants were used to improve genotype accuracy over the imputation-based genotype GWAS of AKI. We additionally rephased these directly genotyped variants using EAGLE (v2.4.1).^23^ GWAS association tests were completed using PLINK 1.90.^26^ We performed both an all ancestry GWAS and an analysis limited to participants of EA. Ten PCs of ancestry were generated with the SNPRelate R package v3.7 at an LD pruning threshold of 0.3.^27^ These PCs were adjusted to control for population stratification. EA was identified on visual inspection of the plotted clusters and selection of the Europeans in the region PC1 <0 and PC2 <0.012.

### In Silico Analysis

We sought evidence for differential expression of *dispatched RND* *transporter family member 1 (DISP1)* and *toll-like receptor 5 (TLR5)* using single-cell RNAseq data from the KPMP.^28^ Single-cell RNAseq was performed in kidney biopsy tissue from 12 patients with AKI and living donor biopsy tissue from 18 healthy donors. Living donor biopsies were obtained before perfusion and before placement in the recipient. The characteristics of the living donor cohort have been described previously.^29^ Methods for tissue processing and RNAseq analyses have been previously published.^30^ We compared *DISP1* and *TLR5* expression between AKI and no AKI biopsy tissue on the basis of a Bonferroni-corrected *P* value adjusted for all the features in the analysis. We also tested whether the variants identified in the intergenic region between *DISP1 and TLR5* were expression quantitative trait loci (eQTL) in the genotype-tissue Expression (GTEx) Portal.^31^ In GTEx, we queried data from 73 participants for whom the results from kidney tissue existed. We also queried the NephQTL database, which includes 187 human kidney samples from patients with nephrotic syndrome in the Nephrotic Syndrome Study Network cohort.^32^ We looked for overlap between the variants with the strongest associations and epigenetic regulatory annotations in the University of California Santa Cruz (UCSC) Genome Browser.^33^

## Results

### Characteristics of Patient Populations

**Figure 1:**
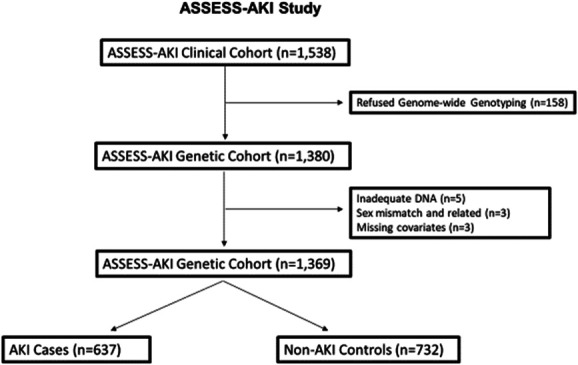
**Patient flow.** One thousand three hundred and sixty-nine participants were included in ASSESS-AKI genetic cohort with 637 AKI cases and 732 non-AKI controls. ASSESS-AKI, Assessment, Serial Evaluation, and Subsequent Sequelae of AKI.

Figure 1 presents the selection of the analytic cohort. We identified 637 cases and 732 controls from the ASSESS-AKI population that consented to genome-wide genotyping. Table 1 summarizes the characteristics of the AKI and non-AKI participants. The participants with AKI had higher rates of inpatient incident dialysis (3.6% versus 0%) and lower eGFR at 3 months (66.7 versus 73.9 ml/min per 1.73 m^2^). Principal Components Analysis (PCA) analysis identified 500 cases and 612 controls as EA and 137 cases and 120 controls who did not cluster with EA participants. See Supplemental Figure 1 for a plot of the PCA and self-reported ancestry.

**Table 1:** Patient characteristics in Assessment, Serial Evaluation, and Subsequent Sequelae of AKI cohort

Characteristics	AKI (*n*=637)	Non-AKI (*n*=732)
**Center, *n* (%)**		
Vanderbilt University	173 (27)	199 (27)
Kaiser permanente, California	133 (21)	181 (25)
Yale	133 (21)	139 (19)
University of Washington, Seattle	198 (31)	213 (29)
Female, *n* (%)	209 (33)	308 (42)
Age, yr, mean (SD)	68.5 (13.1)	70.0 (13.2)
**Race, *n* (%)**		
White	512 (80)	619 (84)
Black	87 (14)	71 (10)
Other	38 (6)	42 (6)
**Comorbidities, *n* (%)**		
Hypertension	499 (78)	501 (68)
CKD	245 (39)	275 (38)
Diabetes	301 (49)	228 (33)
**AKI Risk Factor, *n* (%)**		
Major surgical procedure	255 (40)	380 (52)
Acute heart failure	52 (8.2)	14 (1.9)
Acute myocardial infarction	26 (4.1)	19 (2.6)
Sepsis	107 (17)	26 (4)
Treated in ICU during index admission, *n* (%)	466 (73)	442 (60)
Baseline eGFR, ml/min per 1.73 m^2^, mean (SD)	67.9 (25.7)	71.7 (24.4)
**KDIGO AKI Stage, *n* (%)**		
Stage 1	461 (72.3)	—
Stage 2	95 (14.9)	—
Stage 3	81 (12.7)	—
**Outcomes**		
Acute dialysis, *n* (%)	23 (3.6)	0 (0)
3-mo eGFR, ml/min per 1.73 m^2^, mean (SD)	66.7 (26.9)	73.9 (24.4)

ICU, intensive care unit; KDIGO, Kidney Disease Improving Global Outcomes.

### GWAS Analysis in ASSESS-AKI

**Figure 2:**
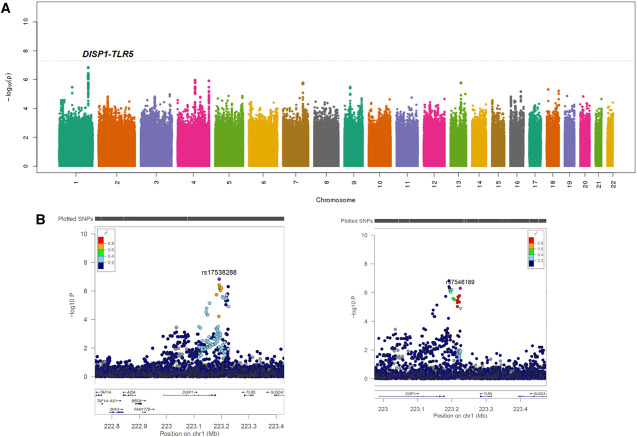
**Manhattan plots generated from genome-wide association study analysis of the AKI cases and controls.** Plots include (A) association results for imputed single-nucleotide polymorphisms (variants) with AKI in the discovery cohort. (B) Genomic locus for the *DISP1-TLR5* top hits, rs17538288 and rs7546189 at higher magnification. LD, r^2^, is indicated relative to the variant with lowest *P* value (purple marker) by colored scale. Notice that the top two *DISP1-TLR5* hits are not in LD with each other (r^2^=0.24). Genes located near each locus are displayed in the bottom panels. *DISP1*, dispatched resistance-nodulation-division (RND) transporter family member 1; LD, linkage disequilibrium; *TLR5*, toll-like receptor 5.

Association testing revealed no variants that satisfied the Bonferroni-corrected genome-wide threshold of *P* < 5×10^−8^. The lambda genomic inflation factor on the basis of a median chi-square was estimated at approximately 1.01 in the all ancestry and the EA-stratified analyses, indicating negligible variation in population structure between cases and controls. The quantile–quantile plot showed overall adherence to expected *P* values (Supplemental Figure 2). Association testing demonstrated 45 variants at nine loci for AKI with *P* < 5×10^−6^ in the full cohort with all ancestries (Figure 2A for Manhattan Plot and Supplemental Excel File 1). Our *P* value threshold for suggestive association was selected based on prior research that a threshold in this range might allow discovery of disease causing variants in smaller sample sizes, while limiting the number of false positive results.^34,35^ Representative variants from each loci were selected based on lowest *P* value and independence of linkage disequilibrium (LD) (r^2^<0.3). Of note, there were two variants selected in the intergenic region between *DISP1* and *TLR5* genes that showed strong association with AKI and low LD (r^2^=0.24 and D′=0.68 in all ancestry and r^2^=0.22 and D′=0.59 in EA). Of the nine top variants at nine different loci, five of the variants were associated with an increased risk of AKI, while four of the nine variants were associated with a decreased risk (Table 2). All nine of the variants were also associated with the severity of AKI (Supplemental Table 1). We also evaluated the association of the top two variants with Stage 1 AKI to no AKI and severe AKI (Stages 2 and 3) compared with no AKI. We found that both the top two variants were nominally associated with severity of AKI (Supplemental Table 2).

**Table 2:** Summary of suggestive imputed variant associations with AKI in Assessment, Serial Evaluation, and Subsequent Sequelae of AKI for joint all ancestry and stratified to European American analysis

Variant	Chr	Position	Gene	MA	MAF	EA MAF	Genotyped	Imputation Quality (r^2^)	All Ancestry OR (95% CI)	All Ancestry *P*^a^	EA OR (95% CI)	EA *P*^a^
rs17538288	1	223192017	*DISP1-TLR5*	A	0.439	0.429	Imputed	0.97	1.54 (1.31 to 1.81)	1.47E-07	1.50 (1.25 to 1.79)	8.49E-06
rs7546189	1	223224864	*DISP1-TLR5*	T	0.347	0.349	Imputed	0.96	1.54 (1.3 to 1.82)	4.85E-07	1.62 (1.35 to 1.96)	3.39E-07
rs80052123	1	89493224	*GBP3* (5′)	A	0.143	0.152	Direct	1.0	1.71 (1.36 to 2.15)	3.41E-06	1.67 (1.31 to 2.13)	3.95E-05
rs6533107	4	104706914	*TACR3* 5′	A	0.352	0.374	Imputed	0.97	0.66 (0.56 to 0.78)	1.09E-06	0.65 (0.54 to 0.78)	4.38E-06
rs9998646	4	188592759	*LINC02492*	C	0.441	0.447	Imputed	0.99	0.68 (0.59 to 0.80)	1.22E-06	0.67 (0.56 to 0.79)	4.10E-06
rs72607731	7	124916848	*LOC101928283*	T	0.237	0.259	Imputed	0.97	1.59 (1.32 to 1.92)	1.65E-06	1.53 (1.25 to 1.88)	3.12E-05
rs1368999	9	28879870	*LINGO2*	C	0.492	0.54	Imputed	0.98	0.67 (0.57 to 0.80)	3.32E-06	0.63 (0.53 to 0.76)	6.10E-07
rs4414368	13	83974202	Gene Desert	T	0.178	0.123	Imputed	0.99	0.58 (0.46 to 0.72)	1.64E-06	0.62 (0.47 to 0.82)	0.0006643
rs9945894	18	9083721	*NDUFV2* 5′	C	0.077	0.075	Imputed	0.98	2.03 (1.50 to 2.75)	4.83E-06	1.90 (1.36 to 2.65)	0.000163

variant, single-nucleotide polymorphism; Chr, chromosome; MA, minor allele; MAF, minor allele frequency; EA, European American; OR, odds ratio; CI, confidence interval; *DISP1*, dispatched resistance-nodulation-division (RND) transporter family member 1; *TLR5*, toll-like receptor 5.

a *P* value and odds ratio after adjustment for patient's age, sex, diabetes, hypertension, enrollment site, and first ten principal components of ancestry in cases and controls.

The variant demonstrating the strongest association with AKI was rs17538288 which is a noncoding intergenic variant between *DISP1* and *TLR5* genes. The minor allele of this variant is associated with an increased risk of AKI (OR, 1.54; 95% CI, 1.30 to 1.81; *P* = 1.47×10^−7^). The next strongest association was observed with rs7546189, which is also in the intergenic region between *DISP1* and *TLR5* and is 33 kilobases from rs17538288. Variant rs7546189 is associated with an increased risk of AKI (OR, 1.54; 95% CI, 1.3 to 1.82; *P* = 4.85×10^−7^). When we restricted participants to EA only (500 cases and 612 controls), rs17538288 (OR, 1.50; 95% CI, 1.25 to 1.79; *P* = 8.48×10^−6^) and rs7546189 (OR, 1.62; 95% CI, 1.35 to 1.96; *P* = 3.39×10^−7^) remained strongly associated with AKI. In the non-EA participants (137 cases, 120 controls), rs17538288 remained nominally significant (OR, 2.06; 95% CI, 1.34 to 3.22; *P = 0.0012*) and rs7546189 did not (OR, 1.29; 95% CI, 0.81 to 1.87; *P = 0.33*).

### *DISP1*-*TLR5* Variants Direct Genotyping Concordance with Imputation

Both top variants were imputed on the Multiethnic Global Array chip, and we directly genotyped rs17538288 and rs7546189 *via* Taqman-based RT-PCR to validate the imputation results. Direct genotypes of rs17538288 were highly concordant with the imputed results (1314/1353; approximately 97.12% concordance). Direct genotypes of rs7546189 were also highly concordant with the imputed genotypes (1289/1334; approximately 96.62% concordance). We updated the discordant imputed genotypes with the RT-PCR results for *post hoc* analyses. The resulting association statistics improved slightly for rs17538288 (OR, 1.55; 95% CI, 1.32 to 1.82; *P = 9.47*×*10*^*−8*^) and remained similar for rs7546189 (OR, 1.53; 95% CI, 1.30 to 1.81; *P = 4.60*×*10*^*−7*^).

### Compound Heterozygosity and Variant Association Independence Analysis for Haplotypes Near the *DISP1-TLR5* Genes

To identify the allele burden association of these two variants (rs17538288 and rs7546189), phased haplotype analysis showed that all four possible two-variant allele haplotype combinations are commonly present (Supplemental Tables 3 and 4). Owing to this, we tested whether the combination of these two variants in a compound heterozygosity association analysis strengthens estimates for AKI. Considering the directly genotyped data, we found that logistic regression tests modeling the combined burden of minor alleles for each variant showed stronger associations for AKI (*P value = 1.77*×*10*^*−9*^) than each variant independently (Table 3). In addition, testing the residuals of one variant's association with AKI with the other variant led both variants to remain significant (*P* values = 0.001 and 0.0002) (Supplemental Figure 3 and Supplemental Table 5). We also evaluated the association of these two variants with the risk of AKI stratified by whether patients had surgery as their primary risk factor for AKI. We found that both variants maintained nominal significance for the outcome of AKI stratified by surgery (Supplemental Table 6).

**Table 3:** Total burden model of minor alleles for *dispatched RND transporter family member 1 toll-like receptor 5* variants, rs17538288 >A and rs7546189 >T.

Number of Minor Alleles, (Number of Patients)	0 (*n*=318)	1 (*n*=318)	2 (*n*=449)	3 (*n*=178)	4 (*n*=100)	Risk of AKI (OR, 95% CI)	*P* Value
AKI, *n* (%)	119 (37%)	136 (43%)	213 (47%)	102 (57%)	67 (67%)	1.34 (1.22 to 1.48)	1.77×10^−9^
No AKI, *n* (%)	205 (63%)	182 (57%)	236 (53%)	76 (43%)	33 (33%)	—	—

Zero designates homozygous major allele for both variants, and four designates homozygous minor allele for both variant. RND, resistance-nodulation-division; OR, odds ratio; CI, confidence interval.

### Functional Annotation of Top-Performing Variants from ASSESS

**Figure 3:**
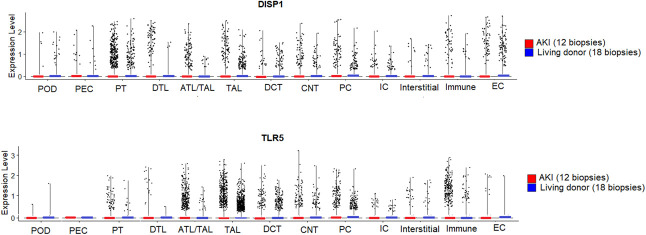
**Single-cell RNA sequencing in living donor (LD) (*n*=18) and AKI (*n*=12) kidney biopsy specimens demonstrates *DISP1* and *TLR5* gene expression in multiple cell lines.** *DISP1* gene expression is significantly upregulated in PT (adjusted *P* = 3.9×10^−2^) and TAL cell types (adjusted *P* = 8.7×10^−3^) in AKI compared with no AKI, while *TLR5* expression is downregulated in TAL cell types (adjusted *P* = 4.9×10^−30^) in AKI compared with no AKI. ATL, ascending thin limb of the loop of Henle; CNT, connecting tubule; DCT, distal convoluted tubule; *DISP1*, dispatched RND transporter family member 1; DTL, descending thin limb of the loop of Henle; EC, endothelial cells; IC, intercalated cells; PC, principal cells; POD, podocytes; PT, proximal tubular epithelial cells and 1–3 segments; TAL, thick ascending limb of the loop of Henle; *TLR5*, toll-like receptor 5.

Demographics and kidney function for the cohort of healthy living donor (*n*=18) and AKI (*n*=12) participants can be found in the supplement (Supplemental Table 7). Examination of single-cell RNA sequencing analyses from kidney biopsies completed in hospitalized patients with AKI through the KPMP^19^ initiative demonstrated that *DISP1* and *TLR5* were expressed in multiple kidney cell types, including proximal tubular epithelial cells and thick ascending limb of the loop of Henle (TAL) (Figure 3). In comparison with kidney tissue from healthy living donor, AKI biopsies showed increased *DISP1* expression in proximal tubular epithelial cells, the primary site of injury in AKI (adjusted *P* = 3.9×10^−2^) and TAL (adjusted *P* = 8.7×10^−3^) (Supplemental Table 7). In addition, AKI biopsies showed decreased expression of *TLR5* in TAL (adjusted *P* = 4.9×10^−30^) (Supplemental Table 8).

Examination of the GTEx and NephQTL also revealed that the two variants, rs17538288 and rs7546189, were eQTLs for *DISP1* gene expression in cardiac tissue (GTEx, *P < 0.0001*, normalized effect size=0.15 and 0.16, respectively) and decreased *HHIPL2* expression in tubulointerstitium kidney tissue (NephQTL, rs17538288, *P = 0.048*, *β*=−0.24). The minor allele of each variant was associated with increased *DISP1* expression. Increased *DISP1* expression was associated with AKI in the KPMP single-cell RNAseq data. By contrast, we found that the minor allele of both variants was associated (*P < 3*×*10*^*−8*^, normalized effect size=−0.24 and −0.29, respectively) with decreased *TLR5* expression in GTEx in cardiac tissue. NephQTL did not show eQTL evidence for either variant with *DISP1* or *TLR5* in *N*=166 tubulointerstitium kidney tissue participants. Decreased *TLR5* expression was associated with AKI in the single-cell RNAseq KPMP analyses (Supplemental Figures 4 and 5). Further inspection of variants in the UCSC Genome Browser^33^ revealed epigenetic regulatory functional annotations in multiple tissues and cell lines. The results can be found in the Supplemental Online text (Supplemental Figure 6 and Supplemental Table 9).

### Replication of Variants from Two Prior AKI GWAS Studies

Review of two previous reported GWAS of AKI^7,8^ failed to reveal any shared variants or genetic loci between the literature and the top-performing variants in ASSESS-AKI. None of the previously reported 22 variants were associated with the development of AKI in ASSESS-AKI (nominal *P* < 0.05) with a similar direction of effect. Because the prior two AKI GWAS studies restricted to persons of EA, we tested the association of prior variants with AKI in persons of EA in ASSESS-AKI and were again unable to detect these previously reported associations (Supplemental Table 10).

## Discussion

We identified nine variants with associations at a level suggestive of being susceptibility loci for AKI risk. The two variants most strongly associated with AKI mapped to the *DISP1-TLR5* locus. Both variants were associated with an increased risk of AKI that reached genome-wide significance in a *post hoc* compound heterozygous analysis. Of note, the minor alleles of both variants were eQTLs for *DISP1*, with the minor allele of each variant associated with increased *DISP1* expression in GTEx. Increased *DISP1* gene expression was present in kidney biopsies from patients with AKI compared with healthy donors. The minor alleles of both variants were also eQTLs for *TLR5* and were associated with reduced *TLR5* expression in GTEx. Reduced *TLR5* gene expression was present in kidney biopsies from patients with AKI compared with healthy donors. Thus, the direction of variant-modulated gene expression and the relationship to risk for AKI may be in opposite directions for the two proximal genes with increased *DISP1* expression and decreased *TLR5* expression associated with risk for AKI.

The gene *DISP1* is involved in sonic hedgehog signaling, and microdeletions of this gene have been associated with developmental delay and dysmorphic features.^36^ In experimental models, sonic hedgehog signaling can accelerate fibrosis postkidney injury and contribute to the development of CKD after AKI.^37,38^ The gene *TLR5* plays a fundamental role in pathogen recognition and activation of innate immune responses, and *TLR5* is highly expressed in the kidneys. Investigators demonstrated that a *TLR5* agonist attenuated kidney injury in a murine model of radiation-induced renal failure and ischemia/reperfusion-induced renal failure through decreased accumulation of reactive oxygen species.^39,40^ This background knowledge supports that the variants, rs17538288 and rs7546189, may influence the risk of AKI through *DISP1* and/or *TLR5* signaling. Because these two variants have low correlation and because the minor allele frequency is approximately 10% different, we completed compound heterozygosity analyses. In these analyses, we demonstrated that the combination of variants strengthens the association with AKI. GTEx data show increased expression of *DISP1* in cardiac tissue and decreased expression of *TLR5* in cardiac tissue in association with rs17538288 and rs7546189. The KPMP single-cell RNAseq results additionally support a differential role for *DISP1* and *TLR5* with risk for AKI. In kidney biopsies from patients with AKI, *DISP1* expression was significantly upregulated, whereas *TLR5* expression was reduced compared with healthy donor biopsies. We hypothesize that upregulation of *DISP1* may lead to hedgehog-mediated effects on kidney tubule regeneration and healing and that downregulation of *TLR5* leads to augmented kidney injury. We also found that the variants identified were distinct from previously reported variants in the *TLR5* gene that were associated with pneumonia susceptibility.^41–43^

Participants in the ASSESS-AKI Study comprise a multiethnic population, and our primary discovery analysis was completed in all persons who agreed to genetic testing in ASSESS-AKI. In sensitivity analyses, we evaluated differences in variant AKI risk on the basis of ancestry. Compared with the all ancestry analysis, we found that rs17538288 had an attenuated risk for AKI in the EA-only population, while the risk of AKI was higher for rs7546189 in the EA-only population. These findings suggest that genetic ancestry may influence the risk of AKI, a complex polygenic trait.^44^ Further studies are necessary to determine whether ancestry is a surrogate for unmeasured or unknown environmental exposures that may influence the risk of AKI.^45–47^

Two prior AKI GWAS studies have been published that have only included patients of EA and mostly postcardiac surgery.^7,8^ We were unable to replicate prior published variants associated with AKI, and this is a challenge in the field.^48,49^ Potential reasons for the lack of replication may be due to the heterogeneity in the AKI clinical syndrome and lack of power due to small sample size.^50,51^ Combining patients with different underlying risk, pathophysiology, and outcomes may dilute the signal to identify individual variants. For example, ASSESS-AKI included a general hospitalized population with a distribution of various AKI risk factors, such as sepsis, surgery, and medications.

Strengths of ASSESS-AKI include the enrollment across multiple centers, the inclusion of participants with diverse ancestry, and the prospective matched cohort study design of the facilitated study of genomic risk of AKI independent of several matching covariates. Second, ASSESS-AKI included a general hospital population at risk for AKI with a mandated baseline serum creatinine measurement before hospitalization. Often in hospitalized patients, an outpatient baseline serum creatinine value is lacking, and thus, the severity and timing of AKI are unknown. Misclassification of AKI status due to a lack of a baseline serum creatinine could dilute potential statistical signals. For example, if a patient carrying a high-risk genetic variant presents to the hospital with AKI but is unrecognized due to a lack of an outpatient baseline, then this patient may be classified as a control, attenuating any potential association signal.

We acknowledge a number of limitations. The main limitation is the sample size, limiting the power to detect variants of small effect sizes or lower population frequencies. Another limitation is that we lacked a second cohort with similar inclusion criteria and genotyping to test replication of top variants.

In summary, we describe the results of a GWAS of AKI with case–control matched participants designed to reduce heterogeneity. No variants reached genome-wide significance. We report two novel variants adjacent to the *DISP1* and *TLR5* genes suggestively associated with AKI susceptibility. Both variants warrant further study on the basis of *post hoc* independence tests, lack of LD, and the increased strength of association when these two *DISP1-TLR5* regulatory variants are considered as compound heterozygotes. Independent evaluation of NephQTL, GTEx, and KPMP single-cell RNAseq data of kidney biopsies supports the interpretation of these variants as being involved in the etiology of AKI. Other independent studies should aim to validate our findings, including functional studies to evaluate the AKI loci reported.

## Disclosures

P.K. Bhatraju reports the following: Research Funding: Roche Diagnostics. V.M. Chinchilli reports the following: Advisory or Leadership Role: Allergan; Astra-Zeneca; Biohaven; Jannsen; Regeneron; Sanofi. S.G. Coca reports the following: Consultancy: 3ive, Axon, Bayer, Boehringer Ingelheim, Nuwellis, Renalytix, Reprieve Cardiovascular, Takeda, Vifor; Ownership Interest: pulseData, Renalytix; Research Funding: ProKidney, Renal Research Institute, Renalytix, XORTX; Patents or Royalties: Renalytix; and Other Interests or Relationships: Associate Editor for *Kidney360*, Editorial Boards of *JASN*, *CJASN*, Kidney International. A.X. Garg reports the following: Research Funding: Astellas, Baxter; Advisory or Leadership Role: Currently on the Editorial Boards of Kidney Int and AJKD; and Other Interests or Relationships: Served as the Medical Lead Role to Improve Access to Kidney Transplantation and Living Kidney Donation for the Ontario Renal Network (government funded agency located within Ontario Health). This position ended October 2022. A.S. Go reports the following: Research Funding: Amarin Pharmaceuticals, Bristol MeyersSquibb, CSL Behring, Janssen Research and Development, Novartis, Pfizer. J. Himmelfarb reports the following: Consultancy: Maze Therapeutics; Ownership Interest: Kuleana Technology, Inc.; Research Funding: Aurinia Pharmaceuticals; Honoraria: various academic institutions for invited lectures; Patents or Royalties: Patents held and patents pending owned by the University of Washington; Advisory or Leadership Role: *CJASN* (Ed board); BMC Medicine (Ed. Board), Nature Reviews Nephrology (Advisory Board); and Other Interests or Relationships: Research grant support from Northwest Kidney Centers; Founder, CEO, President and equity holder, Kuleana Technology, Inc. T.A. Ikizler reports the following: Consultancy: Fresenius-Kabi, Nestle; Research Funding: NIDDK; Veterans Affairs; Honoraria: Fresenius-Kabi, Nestle; and Advisory or Leadership Role: Kidney International. G.P. Jarvik reports the following: Ownership Interest: literally hundreds managed by a professional manager and not chosen by me or my spouse; and Advisory or Leadership Role: I was the 2022 Past President of the American Society of Human Genetics. J.S. Kaufman reports the following: Consultancy: National Kidney Foundation; Otsuka Pharmaceutical; Ownership Interest: Amgen; Advisory or Leadership Role: Digestive and Kidney Diseases, National Institute of Diabetes, paid Steering Committee Chair; and Other Interests or Relationships: Associate Editor, American Journal of Kidney Disease. P.L. Kimmel reports the following: Employer: National Institute of Diabetes and Digestive Kidney Diseases (NIDDK); Ownership Interest: As a Federal Employee at NIDDK, my holdings are reviewed each year for potential conflict of interest. At this time my only stock holding related in any fashion to health care is CVS; Patents or Royalties: Elsevier: Royalties for co-editing Chronic Renal Disease and Psychosocial Aspects of Chronic Kidney Disease; Advisory or Leadership Role: Unpaid member of Board of Directors of Academy of Medicine of Washington, DC; and Other Interests or Relationships: Co-Editor, Chronic Renal Disease Academic Press; Co-Editor, Psychosocial Aspects of Chronic Kidney Disease. Academic Press; Royalties. K. Kiryluk reports the following: Consultancy: Calvariate, HiBio; and Research Funding: Aevi Genomics, AstraZeneca, Bioporto, Vanda, Visterra. S. Menez reports the following: Research Funding: RenalytixAI. A.S. Naik reports the following: Consultancy: CareDX—External Advisor; and Honoraria: CareDx. P.M. Palevsky reports the following: Consultancy: Janssen Research & Development, LLC; and Advisory or Leadership Role: National Kidney Foundation: Past President, Member, Scientific Advisory Board; Renal Physicians Association: Member, Quality, Safety and Accountability Committee; Quality Insights Renal Network 4: Chair, Medical Review Board; UpToDate: Section Editor, RAcute Kidney Injury; Journal of Intensive Care Medicine: Member, Editorial Board. M.R. Palmer reports the following: Employer: Natera, Inc.; and Ownership Interest: Natera, Inc. C.R. Parikh reports the following: Consultancy: Genfit Biopharmaceutical Company; Ownership Interest: Renaltix AI Research Funding: National Institute of Diabetes and Digestive and Kidney Diseases (NIDDK); National Heart, Lung and Blood Institute (NHLBI); and Advisory or Leadership Role: Genfit Biopharmaceutical Company; Renalytix. J.A. Schaub reports the following: Consultancy: Cook Biotech (Spouse); Nuvira (Spouse); Uptodate (Spouse). E.D. Siew reports the following: Ownership Interest: Amazon stock, Apple stock; Patents or Royalties: Author for UptoDate (royalties) and Advisory or Leadership Role: Editorial board of *CJASN*. A. Srivastava reports the following: Consultancy: CVS Caremark; Tate & Latham (Medicolegal consulting); and Honoraria: AstraZeneca; Bayer; FNIH; Horizon Therapeutics PLC. I.B. Stanaway reports the following: Ownership Interest: I have an LLC, Byrell Systems, registered in Washington State that is solely owned by me, Dr. Ian Byrell Stanaway.; and Other Interests or Relationships: ASN Member. F.P. Wilson reports the following: Consultancy: Translational Catalyst, LLC; Ownership Interest: Owner of Efference, LLC; Research Funding: Amgen; Boeringher-Ingelheim; Vifor, Whoop; Advisory or Leadership Role: Editorial Board—American Journal of Kidney Disease; Editorial Board—*Clinical Journal of the American Society of Nephrology*; and Other Interests or Relationships: Medical columnist-Medscape. L.B. Ware reports the following: Consultancy: Akebia, Global Blood Therapeutics, Santhera; Ownership Interest: Virtuoso Surgical; and Research Funding: Boehringer Ingelheim; Genentech. M.M. Wurfel reports the following: Consultancy: Roche Diagnostics; and Research Funding: Roche. All remaining authors have nothing to disclose.

## Supplementary Material

## Supplemental Material

Additional eMethods and eResults.

Supplemental Table 1. Ordinal Regression for KDIGO AKI Severity.

Supplemental Table 2. Association of top two variants and severity of AKI comparing Stage 1 AKI with no AKI and comparing Stage 2 and 3 AKI with no AKI.

Supplemental Table 3. Top Two SNPs adjacent *DISP1-TLR5*, Linkage Disequilibrium Information.

Supplemental Table 4. Case–control counts by *DISP1-TLR5* variants.

Supplemental Table 5. *DISP1-TLR5* Top Two Variants Post Hoc Regression Tests and Condition Adjusted Results with updated RT-PCR validation genotypes.

Supplemental Table 6. Demographics and kidney function in living donor and AKI participants who underwent kidney biopsy for single-cell RNAseq analyses.

Supplemental Table 7. Differential *DISP1* gene expression between living donor and AKI biopsies.

Supplemental Table 8. Differential *TLR5* gene expression between living donor and AKI biopsies.

Supplemental Table 9. Epigenetic Regulatory Information and Cell types from the UCSC Browser.

Supplemental Table 10. European American ASSESS-AKI GWAS Evaluation of SNPs of interest from two prior AKI GWAS.

Supplemental File 1. The 45 associated SNPs with p<5x10^−6^: ASSESS_AKI_GWAS_regression_results.xlsx.

Supplemental File 2. GTEx: Top-Performing SNPs from ASSESS-AKI: GTEx_AKI_GWAS_hits_eQTLs.xlsx.

Supplemental File 3. NephQTL: Top-Performing SNPs from ASSESS-AKI: NephQTL_AKI_GWAS_hits_eQTLs.xlsx.

Supplemental Figure 1. PCA plot in ASSESS.

Supplemental Figure 2. Quantile–quantile AKI GWAS plot in ASSESS.

Supplemental Figure 3. Barplots of the top two *DISP1-TLR5* SNPs.

Supplemental Figure 4. *DISP1-TLR5* SNPs GTEx gene expression.

Supplemental Figure 5. *DISP1* rs17538288 *HHIPL2* NephQTL gene expression.

Supplemental Figure 6. UCSC Genome Browser Screenshot of *DISP1-TLR5* locus with the GeneHancer GH01J223018 epigenetic regulatory site annotated.
