# Supplementary material for: Genome-wide Association Study for AKI
Source: Kidney360. 2023 Jun 5;4(7):870–80. doi: 10.34067/KID.0000000000000175 (PMC10371295; doi:10.34067/KID.0000000000000175)

ONLINE SUPPLEMENTARY MATERIALS

Title: Genome-wide Association Study for Acute Kidney Injury

Authors: Bhatraju PK, Stanaway IB, Palmer MR. et al.

TABLE OF CONTENTS

Additional eMethods and eResults

Table S1. Ordinal Regression for KDIGO AKI Severity

Table S2. Association of top two Variants and severity of AKI comparing Stage 1 AKI to no AKI and comparing Stage 2 and 3 AKI to no AKI

Table S3. Top Two SNPs adjacent *DISP1-TLR5*, Linkage Disequilibrium Information

Table S4. Case control counts by *DISP1-TLR5* variants

Table S5. *DISP1-TLR5* Top Two Variants Post-Hoc Regression Tests and Condition Adjusted Results with updated RT-PCR validation genotypes.

Table S6. Demographics and kidney function in living donor and AKI participants who underwent kidney biopsy for single-cell RNAseq analyses

Table S7. Differential *DISP1* gene expression between living donor and AKI biopsies.

Table S8. Differential *TLR5* gene expression between living donor and AKI biopsies.

Table S9. Epigenetic Regulatory Information and Cell types from the UCSC Browser

Table S10. European American ASSESS-AKI GWAS Evaluation of SNPs of interest from two prior AKI GWAS

Supplemental File 1. The 45 associated SNPs with $p<5x10^{-6}$: ASSESS_AKI_GWAS_regression_results.xlsx

Supplemental File 2. GTEx: Top Performing SNPs from ASSESS-AKI: GTEx_AKI_GWAS_hits_eQTLs.xlsx

Supplemental File 3. NephQTL: Top Performing SNPs from ASSESS-AKI: NephQTL_AKI_GWAS_hits_eQTLs.xlsx

Figure S1. PCA plot in ASSESS

Figure S2. Quantile-quantile AKI GWAS plot in ASSESS

Figure S3. Barplots of the top two *DISP1-TLR5* SNPs

Figure S4. *DISP1-TLR5* SNPs GTEx gene expression

Figure S5. *DISP1* rs17538288 *HHIPL2* NephQTL gene expression

Figure S6. UCSC Genome Browser Screenshot of *DISP1-TLR5* locus with the GeneHancer GH01J223018 epigenetic regulatory site annotated.

Additional eMethods and eResults:

Principal Components

Ancestry was assigned based on these cutoffs of PC1 and PC2. PC1 < 0 and PC2 < 0.012 is European. PC1 > 0.05 and PC2 < 0 is African. PC2 > 0.16 is Asian. All others were classified Admixed.

Admixed	African	Asian	European
90	148	18	1112

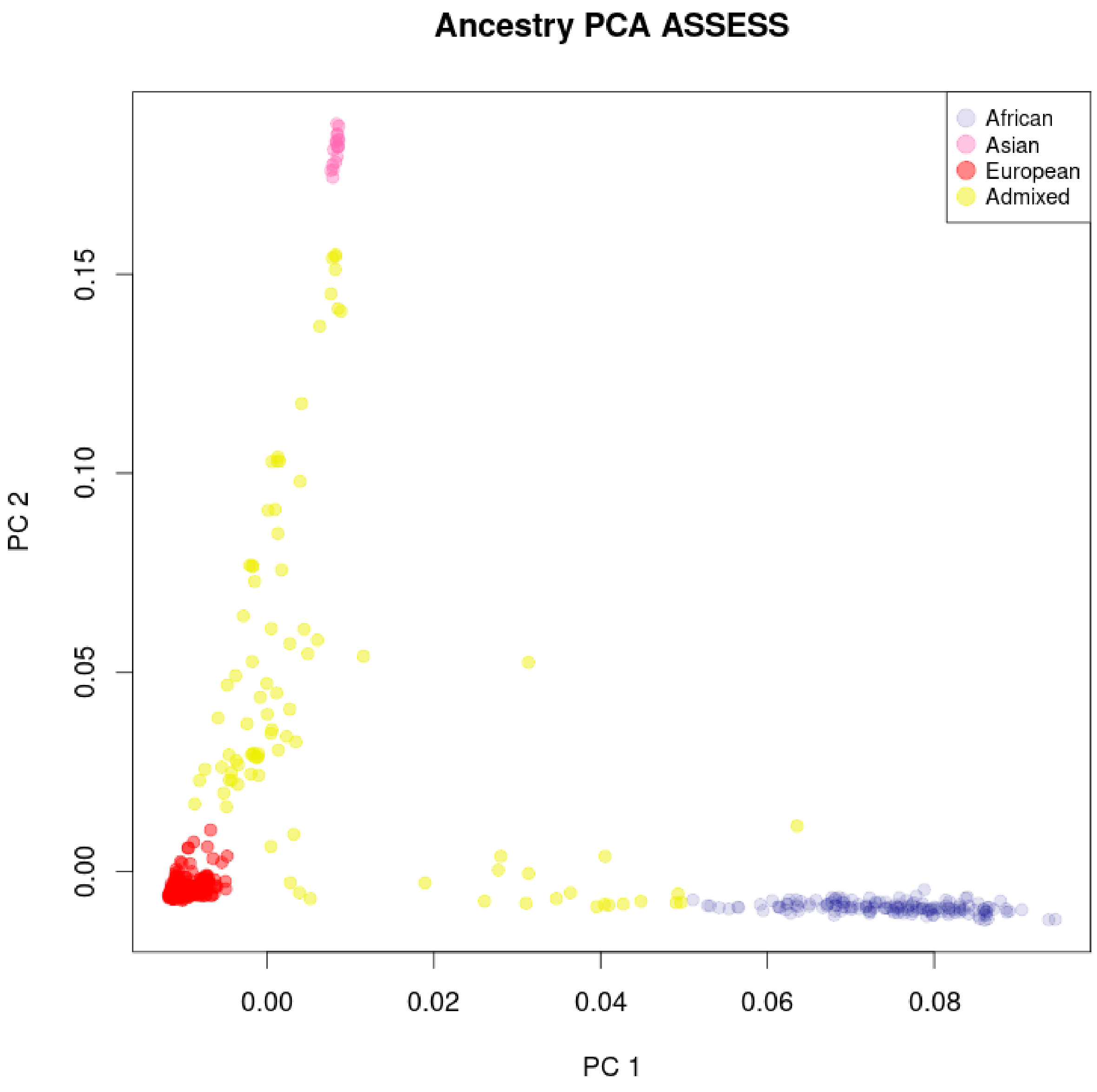

Independence of two SNPs

Additional sensitivity analyses were completed to conclusively show the independence of the two *DISP1-TLR5* adjacent variants, rs17538288 and rs7546189. We made two subsets of the data. We selected the reference homozygous subjects of each of these respective variants. With these subjects, which are now monomorphic at that variant, we then tested the other still polymorphic variant in these two subsets of the data with the confounding effect fixed to zero by the other variant being monomorphic. Both variants are still significant (rs17538288 (232 cases, 352 controls) OR~1.49, 95% CI: 1.13-1.96, *p~0.004* and rs7546189 (169 cases, 263 controls), OR~1.74, 95% CI: 1.14-2.67, *p~0.011*) .

Table S5 Methods

Table S5 presents results of the joint logistic model, recessive models, tests of the residuals or each SNP tested against the association of the other, the two SNPs phased compound heterozygosity (additive 0/1/2), and the summed (0/1/2/3/4) minor allele burden of the two SNPs. Joint logistic and residual models maintained *post-hoc* significance (p-values<0.0045) showing independence of the SNPs in association with AKI. Phased compound heterozygote and the two SNPs allele burden logistic regression tests both showed association to the minor alleles (p-values 10^{-7} ~ 10^{-9}, respectively).

The inpatient dialysis outcome was significant for rs7546189 (OR=2.14 CI 1.13-4.08, p=0.019) and nominally associated with rs17538288 (OR=1.68 CI 0.90-3.19, p=0.108). Compound heterozygote analysis was also significant (OR=2.30 CI 1.19-4.69, p=0.017) as was the two SNP allele burden (OR=1.54 CI 1.07-2.25, p=0.02).

Genomic Context and In-silico eQTLs

We investigated the genomic context and *in-silico* eQTL evidence of the other nominally associated ($p<5 \times 10^{-6}$) loci in the GTEx (https://www.gtexportal.org/) and NephQTL (http://nephqtl.org/) databases. See Supplemental Files 1 (GTEx_AKI_GWAS_hits_eQTLs.xlsx) and 2 (NephQTL_AKI_GWAS_hits_eQTLs.xlsx) complete lists of tissues and association statistics.

The SNP rs80052123 (hg19 chr1:89493224) is in the 5' region of guanylate binding protein 3 (*GBP3*). *GBP3* is expressed in the GTEx kidney cortex data and significantly differentially

expressed in association with this SNP in 21 tissues, the most significant is Adipose Viseral (Omentum) Tissue (NES 0.39, p=6.1 x 10^{-11}). rs80052123 is also associated with differential expression of GBP7 in 12 tissues with the most significant also being Adipose Viseral (Omentum) Tissue (NES 0.45, p=1.1x10^{-11}). The gene KYAT3 is also differentially expressed in association with rs80052123 in Brain Putamen basal ganglia (NES 0.45, p=0.000011) and Brain Caudate basal ganglia (NES 0.4, p=0.000018) tissues. See Supplemental Excel file (GTEx_AKI_GWAS_hits_eQTLs.xlsx) for all tissue results and Figure S5 for a selection of tissue specific GTEx boxplots. In the NephQTL data rs80052123 is associated with differential expression of GBP3 (Beta 0.39, p=0.016) and GBP4 (Beta 0.26, p=0.032) in the Glomerulus (See Figure S6 for boxplots) and GBP3 is suggestive (Beta 0.22, p=0.058) in tubulointerstitium.

The SNP rs17538288 (hg19 chr1:223192017) is in the 3' region of dispatched homolog 1 (Drosophila) (*DISP1*). *DISP1* is significantly (NES 0.15, p=0.000097) differentially expressed in the Left Heart Ventricle tissue in association with rs17538288 (See Supplemental Figure S3 for Boxplots). rs17538288 is also associated with differential expression of the other nearby gene toll-like receptor 5 (*TLR5*) (NES -0.24, p=2.70x10^{-8}) in Left Heart Ventricle tissue and *RP11-452F19.3* (NES -0.16, p=0.000012) in Tibial Artery. In the NephQTL data rs17538288 is associated with differential expression of the nearby *HHIPL2* gene (Beta -0.237, p=0.048) in kidney tubulointerstitium tissue.

The SNP rs7546189 (hg19 chr1:223224864) is also in the 3' region of *DISP1*. *DISP1* is significantly (NES 0.16, p=0.000099) differentially expressed in the Left Heart Ventricle tissue in association with rs7546189 (See Supplemental Figure S3 for Boxplots). rs7546189 is also associated with differential expression of the other nearby gene *TLR5* (NES -0.29, p=3.50x10^{-10}) in Left Heart Ventricle tissue.

The SNP rs6533107 (hg19 chr4:104706914) is in the 5' region of tachykinin receptor 3 (*TACR3*). *TACR3* is expressed in the GTEx kidney cortex data and significantly differentially expressed in association with this SNP in Lung tissue (NES -0.17, p=7.9 x 10^{-5}). See Figure S7 for *TACR3* boxplots. In the NephQTL data rs6533107 is associated with differential expression of the nearby (~360KB) *LINC02428* (Beta 0.26, p=0.018) in kidney tubulointerstitium (Figure S8).

The SNP rs9998646 (hg19 chr4:188592759) is in an intron of *LINC02492*. GTEx shows a significant sQTL with the genomically overlapping *RP11-565A3.1* transcript (NES -0.43, p=5x10^{-7}) in Testis tissue.

The SNP rs72607731 (hg19 chr7:124916848) is in an intron of the ncRNA LOC101928283. The nearby (~133,600 bp) lncRNA gene POT1 antisense RNA 1 (*POT1-AS1*) is differentially expressed in three tissues (Testis, Atrial Heart Appendage, Aorta Artery; NES 0.19, 0.33, 0.28; p=0.000014, 0.000017, 0.000043; respectively). Another nearby (510,770bp) gene G protein-coupled receptor 37 (*GPR37*) is also differentially expressed (NES 0.29, p=8.7x10^{-6}) in Tibial Nerve tissue. The highly expressed in GTEx Kidney Cortex and Medulla lincRNA gene *RP11-807H17.1* is differentially expressed (NES -0.46, p=0.000047) in Brain Cortex tissue. *RP11-807H17.1* is genomically overlapping with *POT1-AS1*. In the NephQTL data rs72607731 is suggestively associated with differential expression of the *C7ORF77* gene (Beta 0.27, p=0.051) in kidney tubulointerstitium.

The SNP rs1368999 (hg19 chr9:28879870) is in an intron of leucine rich repeat and Ig domain containing 2 (*LINGO2*). *LINGO2* is expressed in the GTEx kidney cortex data and significantly differentially expressed in association with this SNP in Nerve Tibial tissue (NES 0.26, p=1.3x10^{-6}). See Figure S9 for rs1368999 GTEx boxplots. In the NephQTL data rs1368999 is associated with differential expression of the nearby (~9kb) gene *MIR873* (Beta 0.312, p=0.0162) in glomerulus (Figure S10). *LINGO2* is genomically overlapping with *MIR873* sharing sequence.

The SNP rs9945894 (hg19 chr18:9083721) is in 5' region of NADH dehydrogenase (ubiquinone) flavoprotein 2 (*NDUFV2*). *NDUFV2* is highly expressed in the GTEx kidney cortex data and significantly differentially expressed in association with this SNP in Testis tissue (NES 0.26 , p=1.8x10^{-6}). See Figure S11 for rs9945894 *NDUFV2* boxplots. In GTEx rs9945894 is also associated with differential expression of the nearby (~250KB) gene twisted gastrulation homolog 1 (*TWSG1)* in 10 tissues (See Supplemental File 2) with the most significant (NES -0.3, p=3.1x10^{-11}) being Esophagus Mucosa.

Among our most associated SNP loci, all showed significant cis eQTL effects except rs4414368 (hg19 chr13:83974202) which is in a gene desert and not associated with differential expression of current GTEx and NepthQTL annotated transcripts. The gene SLIT and NTRK-like family,

member 1 (*SLITRK1*) is 478,855 bp and the lincRNA *LINC00377* is ~2,360,000 bp away toward the centromere.

Table S1 Ordinal Regression for KDIGO AKI Severity

SNP	Chr	Position	Gene	MA	All Ancestry MAF	EA MAF	All Ancestry OR (95% CI)	All Ancestry *P**
rs80052123	1	89493224	*GBP3* 5'	A	0.143	0.152	1.53 (1.24-1.88)	5.39e-05
rs17538288	1	223192017	*DISP1-TLR5*	A	0.439	0.429	1.46 (1.25-1.69)	1.01e-06
rs7546189	1	223224864	*DISP1-TLR5*	T	0.347	0.349	1.45 (1.25-1.70)	1.97e-06
rs6533107	4	104706914	*TACR3* 5'	A	0.352	0.374	0.68 (0.58-0.79)	1.38e-06
rs9998646	4	188592759	Gene Desert	C	0.441	0.447	0.70 (0.60-0.80	1.03e-06
rs72607731	7	124916848	Gene Desert	T	0.237	0.259	1.50 (1.26-1.79)	5.20e-06
rs1368999	9	28879870	*LINGO2*	C	0.492	0.54	0.67 (0.57-0.78)	3.59-07
rs4414368	13	83974202	Gene Desert	T	0.178	0.123	0.58 (0.47-0.72)	8.85e-07
rs9945894	18	9083721	*NDUFV2* TFBS	C	0.077	0.075	1.93 (1.47-2.53)	1.85e-06

SNP – single nucleotide polymorphism, MA – minor allele, MAF – minor allele frequency, OR - odds ratio, CI-confidence interval.

The outcome is modeled as an ordinal variable with (0=No AKI),1=KDIGO Stage 1, 2=KDIGO Stage 2, and 3=KDIGO Stage 3)

Table S2. Association of top two Variants and severity of AKI comparing Stage 1 AKI to no AKI and comparing Stage 2 and 3 AKI to no AKI

Variant	Gene	All Ancestry OR (95% CI)	All Ancestry *P**	Surgery Population OR (95% CI)	P	Non-surgery Population OR (95% CI)	P
rs17538288	***DISP1-TLR5***	**1.54 (1.31-1.81)**	**1.47E-07**	1.39 (1.09 – 1.77)	7.2E-03	1.66 (1.33 – 2.09)	9.18E-05
rs7546189	***DISP1-TLR5***	**1.54 (1.3-1.82)**	**4.85E-07**	1.55 (1.21 – 1.99)	6.2E-04	1.53 (1.21 – 1.93)	3.4E-04

In this analysis, 732 patients had no AKI, 461 had Stage 1 AKI and 176 had Stage 2 and 3 AKI.

Table S3. Top Two variants intergenic of *DISP1* and *TLR5*, Linkage Disequilibrium Information

***DISP1-TLR5* two SNPs, rs17538288 G>A and rs7546189 C>T**		
**R-square =** 0.239113	**D` =** 0.594123	
**Haplotype**	**Frequency**	**Frequency Expectation under LE**
**AT**	0.267680	0.152195
**GT**	0.078894	0.194380
**AC**	0.171460	0.286945
**GC**	0.481966	0.366480

LE = Likelihood Expectation

Table S4. Case control counts by variants updated with RT-PCR genotypes

**rs17538288 -- A Allele**			
**Allele Count**	**0**<br>**(n=432)**	**1**<br>**(n=669)**	**2**<br>**(n=268)**
**AKI, n (%)**	169 (39%)	303 (45%)	165 (62%)
**No AKI, n (%)**	263 (61%)	366 (55%)	103 (38%)
**rs7546189 -- T Allele**			
**Allele Count**	**0**<br>**(n=593)**	**1**<br>**(n=607)**	**2**<br>**(n=169)**
**AKI**	238 (40%)	295 (49%)	104 (62%)
**No AKI, n (%)**	355 (60%)	312 (51%)	65 (38%)

Table S5. Top two variants post-hoc regression tests and condition adjusted results with updated RT-PCR validation genotypes.

Top Variants Models	OR	OR 95% CI	z-value	p-value
**Independent Logistic Models – Additive**				
rs17538288 A	1.55	1.32 – 1.82	5.337	9.47×10^{-8}
rs7546189 T	1.53	1.30 – 1.81	5.042	4.60×10^{-7}
**Independent Logistic Models – Recessive**				
rs17538288 A	2.20	1.66 – 2.94	5.378	7.54×10^{-8}
rs7546189 T	2.07	1.47 – 2.92	3.685	3.35×10^{-5}
**Joint Logistic Model – Additive**				
rs17538288 A	1.37	1.14 – 1.64	3.373	0.000743
rs7546189 T	1.32	1.09 – 1.59	2.873	0.004069
**Independent Logistic Additive Residuals tested in 2nd Linear Regression Models**				
rs17538288 A	1.17	1.08 – 1.28	3.678	0.00024
rs7546189 T	1.16	1.06 – 1.26	3.214	0.00134
**Two Variants Phased Compound Heterozygote Logistic Model – Additive**				
0/1/2	1.59	1.35 – 1.86	5.686	1.30×10^{-8}
**Two Variants Phased Compound Heterozygote Logistic Model – Recessive**				
0/1	1.97	1.54 – 2.54	5.298	1.17×10^{-7}
**Two Variants Summed Minor Allele Burden Logistic Model – Additive**				
0/1/2/3/4	1.34	1.22 – 1.48	6.018	1.77×10^{-9}

Table S5. Association of top two variants with AKI stratified by surgery as primary risk factor for AKI

Variant	Gene	All Ancestry OR (95% CI)	All Ancestry *P**	Surgery Population OR (95% CI)	P	Non-surgery Population OR (95% CI)	P
rs17538288	*DISP1-TLR5*	1.54 (1.31-1.81)	1.47E-07	1.39 (1.09 – 1.77)	7.2E-03	1.66 (1.33 – 2.09)	9.18E-05
rs7546189	*DISP1-TLR5*	1.54 (1.3-1.82)	4.85E-07	1.55 (1.21 – 1.99)	6.2E-04	1.53 (1.21 – 1.93)	3.4E-04

In this sub-group analysis, among patients with surgery, 380 patients had no AKI and 255 had AKI and among patients without surgery 352 had no AKI and 382 had AKI.

Table S6. Demographics and kidney function in living donor and AKI participants who underwent kidney biopsy for single-cell RNAseq analyses

	**Living Donor (n=18)**	**AKI (n=12)**
**Age, average ± SD (range), years**	45.1 ± 10.2 (30-66)	50.4 ± 18.4 (23-78)
**Female, n (%)**	11 (61)	8 (73)
**Race, n (%)**		
White	16 (88)	5 (46)
Black	1 (6)	5 (46)
Other	1 (6)	1 (8)
**Iothalamate GFR, average ± SD (range), ml/min/1.73 m2**	100.6 ± 16.9 (81-144)	-

Table S7. Differential *DISP1* gene expression between living donor and AKI biopsies.

Cell Type	Average_logFC	Cells expressing DISP1 in AKI (%)	Cells expressing DISP1 in LD (%)	p-value	adjusted p value
PT	0.0482	3.3	0.019	1.31E-06	3.98E-02
TAL	0.01	1.3	0.028	2.86E-07	8.70E-03

Abbreviations; thick ascending limb of the loop of Henle (TAL); proximal tubular epithelial cells (PT)

Adjusted p-value based on a Bonferroni correction using all genes.

Table S8. Differential *TLR5* gene expression between living donor and AKI biopsies.

Cell Type	Average_logFC	Cells expressing *TLR5* in AKI (%)	Cells expressing *TLR5* in LD (%)	p-value	adjusted p value
PT	0.01403	0.7	0.2	8.95e-05	1
TAL	-0.0115	5.1	12.2	1.61e-34	4.91e-30

Abbreviations; thick ascending limb of the loop of Henle (TAL); proximal tubular epithelial cells (PT)

Adjusted p-value based on a Bonferroni correction using all genes.

Table S9. Epigenetic Regulatory Information and Cell types from the UCSC Browser

**rs17538288 chr1:223192017 Minor Allele: A MAF 0.439**	
**Epigenetic Experiment Types with Signals**	
GeneHancer Site GH01J223018	
DNAase1	
POLR2A Chip-seq	
Multiple Histone Modifications	
ChIA-PET	
Open Chromatin	
**Cell lines with Epigenetic Signals at rs17538288**	
**Cell Line**	**Description**
K562	bone marrow, chronic myelogenous leukemia
H1-hESC	H1 human embryonic stem cell line
H7-hESC	undifferentiated embryonic stem cells
GM12878	1000 Genomes lymphoblasts
HeLa	cervix epithelial adenocarcinoma
HUVEC	umbilical vein/vascular endothelium
GM78	metachromatic leukodystrophy
U2OS	bone epithelial
NT2-D1	testis, metastatic lung, epithelial like
Th1	primary Th1 T cells
LNCaP	prostate adenocarcinoma
Osteobl	osteoblasts
Chorion	outermost of two fetal membranes
**rs7546189 chr1:223224864 Minor Allele: T MAF: 0.347**	
**Epigenetic Experiment Types with Signals**	
DNAase1	
Multiple Histone Modifications	
Open Chromatin	
**Cell lines with Epigenetic Signals at rs7546189**	
**Cell Line**	**Description**
K562	bone marrow, chronic myelogenous leukemia
hMEC	Primary Mammary Epithelial
H1-hESC	H1 human embryonic stem cell line
NHLF	Primary Lung Fibroblast, Normal
GM12878	1000 Genomes lymphoblasts
HepG2	liver hepatocellular carcinoma
HUVEC	umbilical vein/vascular endothelium
GM78	metachromatic leukodystrophy
NT2-D1	testis, metastatic lung, epithelial like
U2OS	bone epithelial

Table S10. European American ASSESS-AKI GWAS Evaluation of SNPs of interest from two prior AKI GWAS

				Prior AKI GWAS Effect Size		ASSESS Effect Size	
**Previous AKI GWAS**	**rsID**	**Chr**	**BP**	**B (95% CI) or OR (95% CI)**	**p-value**	**OR**	**P-value**
**Stafford-Smith M. et al. *KI***	rs2352039	1	78819945	10.1 (5.6 – 14.5)	9.78×10^{-6}	0.9504	0.6666
	rs13317787	3	8141952	21.6 (12.1 - 31.1)	9.67×10^{-6}	0.8134	0.4449
	rs10262995	7	33550041	14.3 (8.5 – 20.1)	1.83×10^{-6}	0.9882	0.931
	rs2248098	12	48253356	-7.48 (-4.3 - -10.6)	3.60×10^{-6}	1.059	0.517
	rs1109836	16	57660346	37.6 (23 – 52.2)	5.67×10^{-7}	1.561	0.3084
	rs8086030	18	9750395	7.6 (4.3 – 10.9)	7.33×10^{-6}	1.063	0.5064
	rs8099036	18	9756056	8.5 (5.1 – 11.8)	9.01×10^{-7}	1.039	0.6856
	rs2831026	21	28969040	9.0 (5.4 - 12.5)	1.11×10^{-6}	0.8726	0.169
	rs1551588	21	28982407	11.9 (7.6 – 16.2)	9.14×10^{-8}	0.8013	0.06302
	rs12134263	1	84693204	2.06 (1.52 – 2.79)	3.47×10^{-6}	0.9789	0.8967
	rs1416526	1	217236895	1.75 (1.38 – 2.20)	2.47×10^{-6}	1.005	0.9646
	rs10166390	2	43342608	1.98 (1.43 – 2.73)	3.35×10^{-5}	0.8788	0.3995
	rs62341639	4	185159434	0.61 (0.49 – 0.75)	3.98×10^{-6}	1.004	0.9717
	rs62341657	4	185159538	0.61 (0.49 – 0.75)	3.98×10^{-6}	0.9944	0.9605
	rs10815381	9	6226289	0.61 (0.50 – 0.74)	7.11×10^{-7}	0.8594	0.1475
	rs10975499	9	6230072	0.61 (0.50 – 0.75)	1.21×10^{-6}	0.8762	0.2035

**Zhao B. et al.** ***AJRCCM***	rs10810119	9	14226139	0.63 (0.51 – 0.79)	3.49×10^{-5}	1.044	0.7052
	rs10961436	9	14229179	0.58 (0.45 – 0.75)	4.40×10^{-5}	0.9927	0.9578
	rs10961440	9	14234315	0.62 (0.50 – 0.77)	1.53×10^{-5}	1.17	0.1795
	rs2821525	9	15507286	2.31 (1.62 – 3.38)	2.55×10^{-6}	1.343	0.1791
	rs114950412	9	15520416	2.25 (1.59 – 3.19)	4.80×10^{-6}	1.36	0.1624
	rs148018420	12	16438896	0.017 (0.004 – 0.070)	1.43×10^{-8}	1.189	0.636
	rs56194898	13	111012570	2.64 (1.70-4.12)	1.76×10^{-5}	1.336	0.1403
	rs11570785	14	50862057	0.35 (0.22 – 0.55)	4.00×10^{-6}	0.9724	0.9007
	rs9617814	22	19609943	0.66 (0.54 – 0.80)	2.04×10^{-5}	0.9619	0.6991
	rs10854554	22	19610682	0.65 (0.53 – 0.79)	1.44×10^{-5}	0.8815	0.2285
	rs111732708*	22	43787850	4.37 (2.20 – 8.67)	2.51×10^{-5}	1.181	0.6935
	rs148955421			2.36 (1.65 – 3.38)	2.55×10^{-6}	indel NA	indel NA
	rs35560890			0.67 (0.56 – 0.79)	1.81×10^{-6}	indel NA	indel NA
	rs202139590			4.94 (2.40 – 10.18)	1.45×10^{-5}	indel NA	indel NA

*variant is not in the EA ASSESS-AKI GWAS, but was reported in the All Ancestry ASSESS Results.

Figure S1. PC1 and PC2 plot of the LD pruned (threshold=0.3) SNVs with Self-Reported Ancestry.

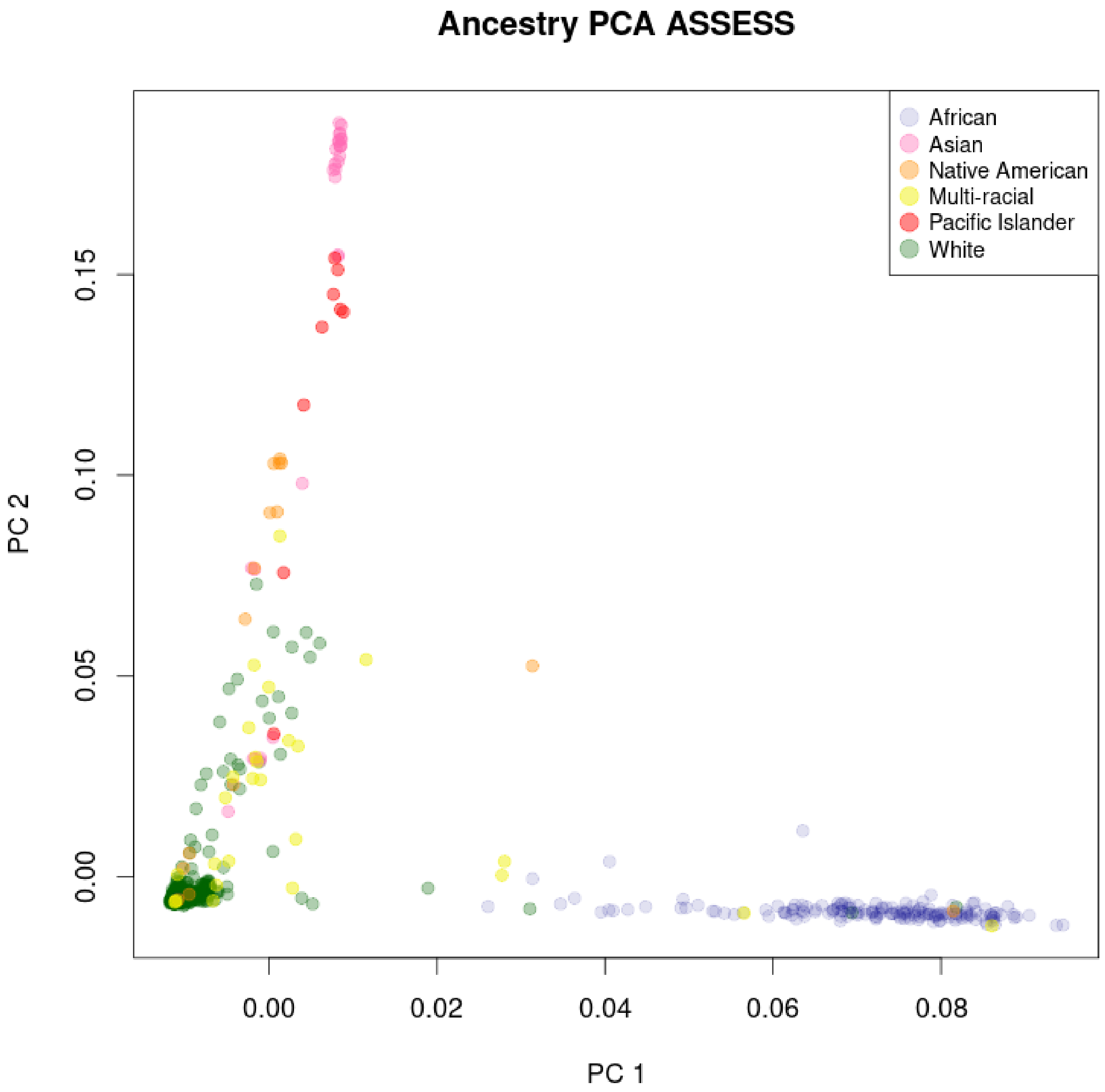

Figure S2. Quantile-quantile plot in ASSESS showing overall adherence to expected *p* values. The genomic inflation factor based on a median chi-square was estimated at 1.01, indicating negligible variation in population structure between cases and controls.

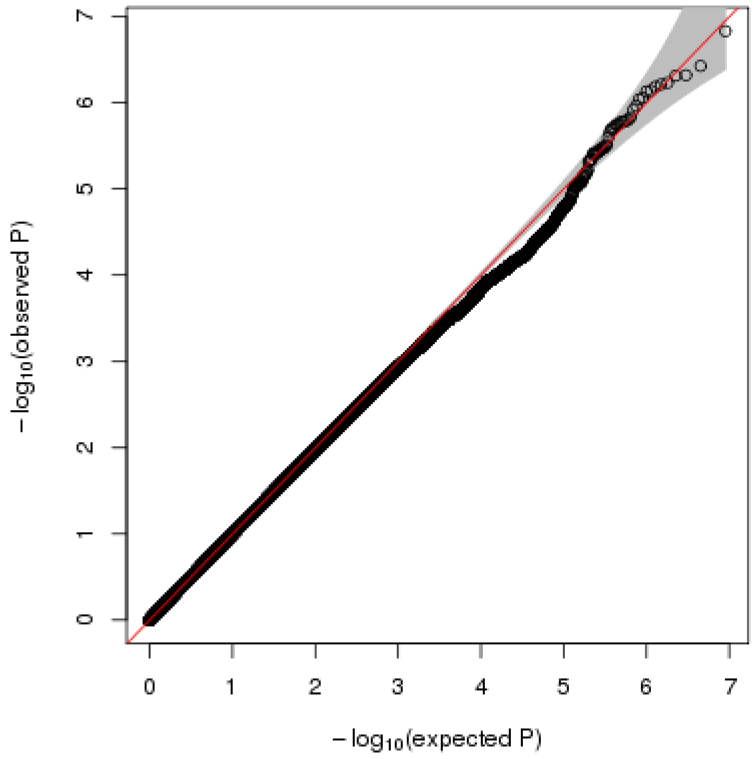

Figure S3. Barplots of the top two *DISP1-TLR5* SNPs displayed by the frequency in AKI cases and controls based on the count of minor alleles after correction of the imputation with the direct genotyping RT-PCR results. The bars labelled as Compound Heterozygote are based on the chromosomal phase of both SNPs and depict that proportion of subjects' that have 0, 1 or 2 minor alleles of either SNP. The denominator for the red bars is the total number of AKI cases (N=637). The denominator for the blue bars is the number of controls (N=732).

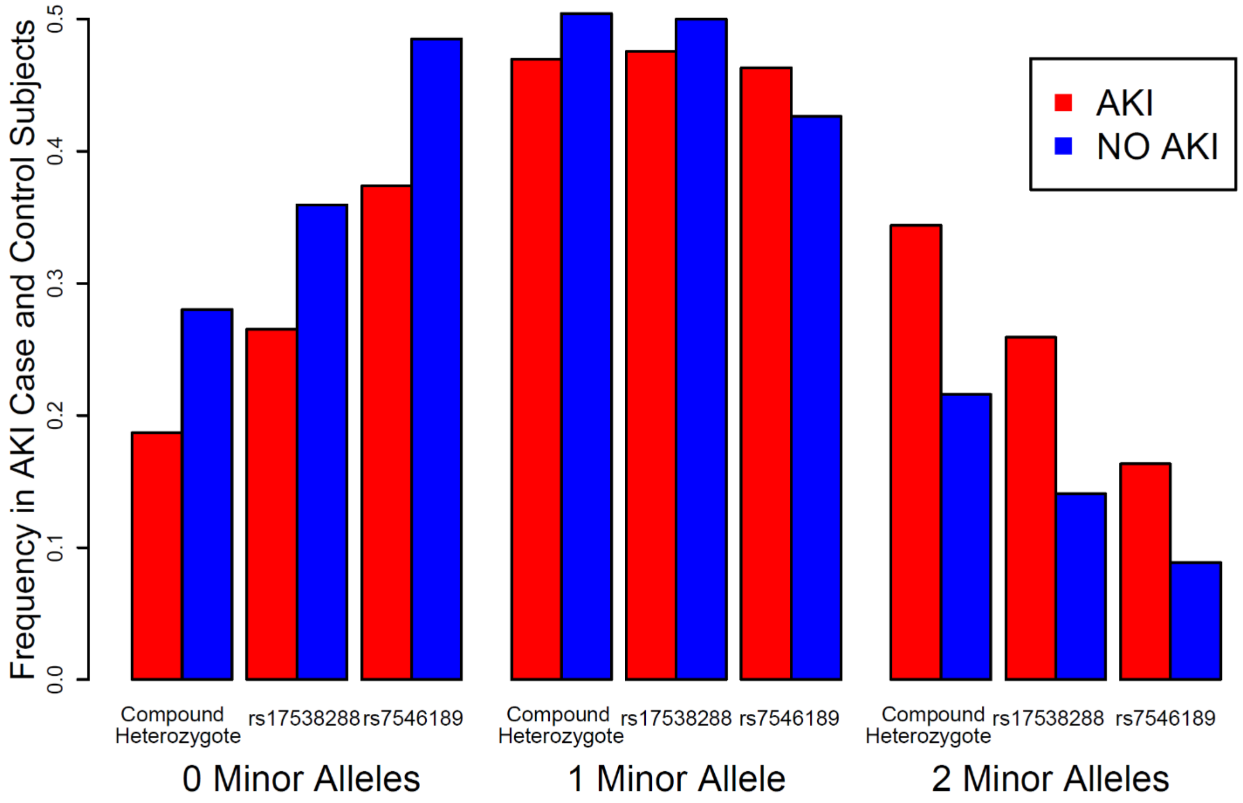

Figure S4. *DISP1* and *TLR5* GTEx gene expression

rs17538288 rs7546189

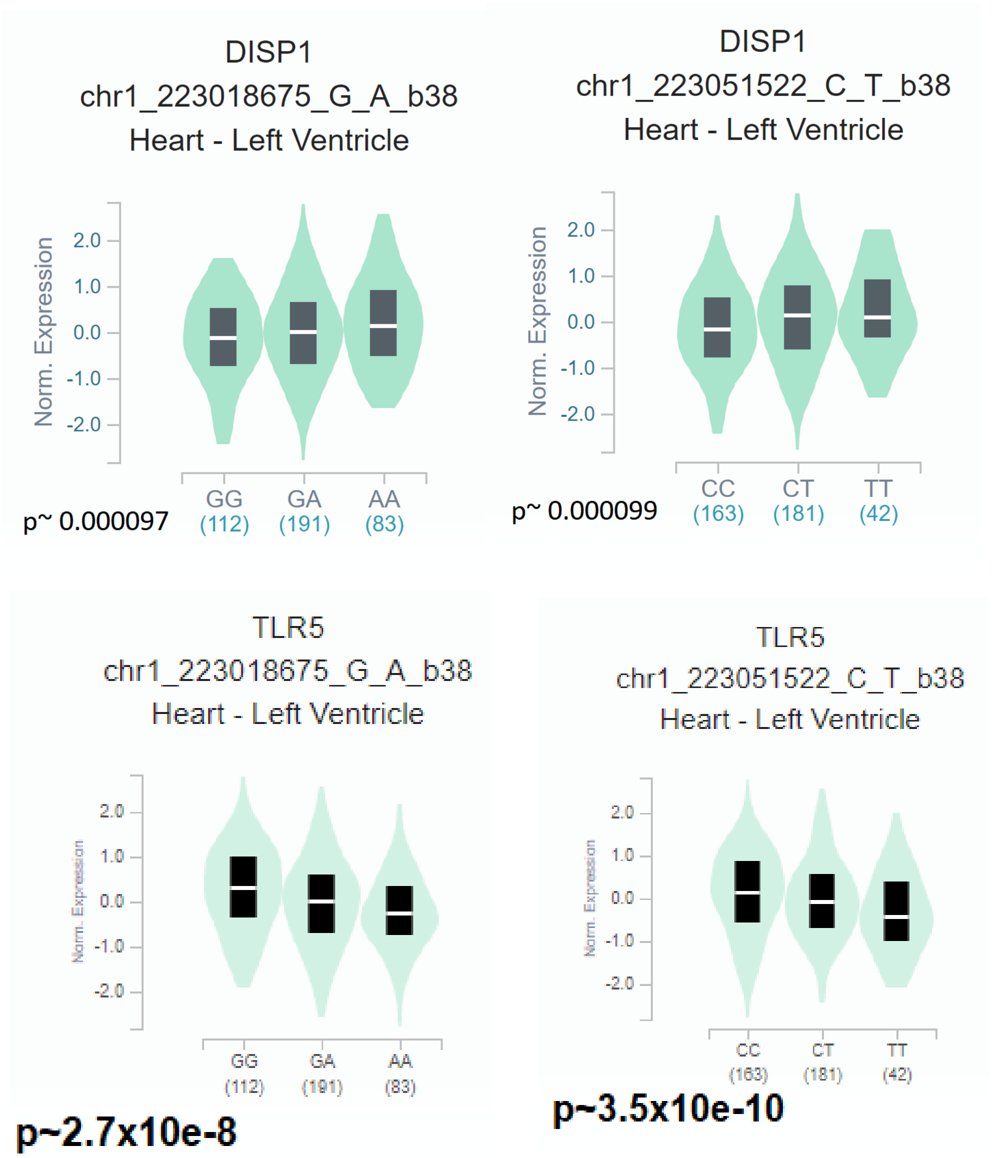

Figure S5. *DISP1* rs17538288 *HHIPL2* NephQTL gene expression

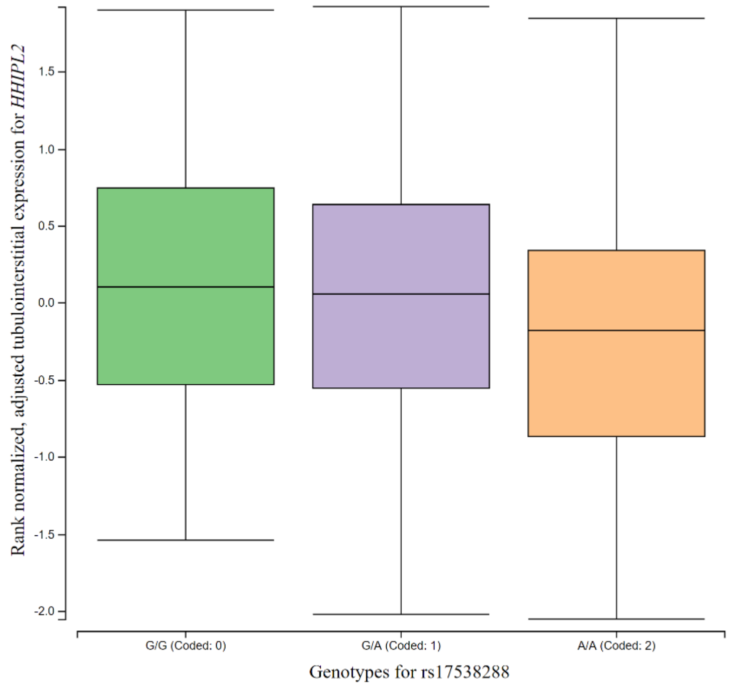

Figure S6. UCSC Genome Browser Screenshot of DISP1 locus with the GeneHancer GH01J223018 epigenetic regulatory site annotated.

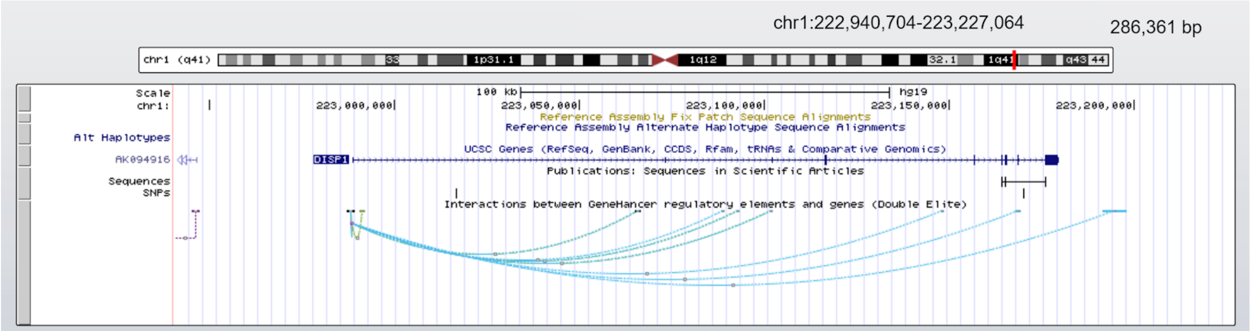

Supplement: Supplementary file 1 [file kidney360-4-0870-s001.pdf]
